# Late Presentation of Fetus in Fetu in a 10‐Year‐Old Boy

**DOI:** 10.1155/crpe/9453323

**Published:** 2026-11-04

**Authors:** Kinza Akbar, Ali Raza, Aden Rehman, Muhammad Wajid Siddique, Tahreem Tariq, Muhammad Riaz ul Haq, Nazila Dalir

**Affiliations:** ^1^ Sahiwal Medical College Sahiwal Pakistan; ^2^ Ameer-ud-Din Medical College Lahore Pakistan; ^3^ School of Medicine St. George’s University St. George’s Grenada sgu.edu

**Keywords:** abdominal mass, child, computed tomography, fetus in fetu, teratoma, ultrasonography, Willis criteria

## Abstract

Fetus in fetu (FIF) is a rare congenital anomaly in which a dysmorphic, parasitic twin is retained within the body of its host twin. It typically presents in infancy, shows a slight male predominance, and is most often located in the retroperitoneum. Differentiating FIF from an abdominal teratoma on imaging is important, as the two entities can closely resemble one another. We report the case of a 10‐year‐old boy who presented with abdominal pain, low‐grade fever, and unintentional weight loss. Antenatal history was unremarkable. Abdominal examination revealed mild distension and left‐sided tenderness. Ultrasonography demonstrated an 8.5 × 10.4 × 11.1 cm mass‐like lesion in the left hypochondrium, and a provisional diagnosis of teratoma versus FIF was considered. Contrast‐enhanced computed tomography (CT) of the abdomen showed a large, well‐defined, heterogeneous mesenteric mass measuring 10.0 × 11.0 × 14.0 cm containing macroscopic fat, dense tooth–like calcifications, and osseous elements with attenuation of up to 1450 Hounsfield units, together with a well‐formed fetal skeleton. Serum tumor markers were within normal limits. A presumptive diagnosis of FIF, supported by imaging and by intraoperative findings that satisfied the Willis criteria, was made at exploratory laparotomy; histopathological confirmation could not be obtained because the specimen was inadequately fixed prior to processing, so a highly differentiated fetiform teratoma cannot be entirely excluded. The child remained well at last follow‐up, about 10 weeks after discharge; longer surveillance is required. This case illustrates that FIF, although classically a diagnosis of infancy, should remain in the differential diagnosis of a complex abdominal mass in an older child, and highlights the role of the Willis criteria, radiological correlation, and intraoperative findings when histopathology is unavailable.

## 1. Introduction

Fetus in fetu (FIF) is a rare condition in which a dysmorphic, parasitic twin is retained within the body of its host twin [1, 2]. It is generally believed to arise from aberrant monozygotic twinning during early embryogenesis, although its precise embryological basis and its relationship to highly differentiated teratomas remain debated [2, 3]. FIF typically presents in infancy, shows a slight male predominance, and is most often located in the retroperitoneum [1, 4]; occasional later presentations, including in adolescence and adulthood, have nonetheless been reported [1, 5]. Clinical features vary with lesion size and location [3]. Cross‐sectional imaging is central to diagnosis, as it can demonstrate calcifications, fat, long bones, or an organized axial skeleton within the mass [1, 3]. A key diagnostic challenge is distinguishing FIF from a mature or fetiform teratoma; an organized vertebral axis with appropriately arranged limbs is considered more supportive of FIF, in keeping with the Willis criteria, although this distinction is not always definitive and ultimately depends on correlating imaging, intraoperative findings, and, where possible, histopathology [3, 6, 7]. We describe a case of FIF in a 10‐year‐old boy presenting with abdominal pain and a large abdominal mass containing osseous and dental elements, illustrating that this rare condition can present outside infancy, and discussing the diagnostic approach when histopathological confirmation cannot be obtained.

## 2. Case Presentation

A previously healthy 10‐year‐old boy presented to the surgical emergency department and was admitted on June 16, 2026, with a 10‐day history of abdominal pain associated with low‐grade fever and unintentional weight loss. There was no history of vomiting, hematemesis, melena, dysuria, hematuria, or lower‐limb weakness.

Antenatal history was unremarkable: the child was born at term by spontaneous vaginal delivery. No antenatal ultrasound had been performed, and a twin gestation was not documented. The parents were nonconsanguineous, and family history was noncontributory.

On general examination, the patient was hemodynamically stable, alert, and chronically ill‐appearing, with mild pallor. Pulse rate was 98 beats/min, and temperature was 99.6°F (37.6°C). The abdomen was soft, mildly distended, and tender on the left side, without a palpable mass. Bowel sounds were active, with normal passage of stool and flatus. Digital rectal examination showed increased anal tone; the examining finger was stained with stool, and no mass was palpable.

Doppler ultrasonography and contrast‐enhanced computed tomography (CT) (CECT) of the abdomen had been performed prior to admission, on June 15, 2026. Ultrasonography demonstrated an 8.5 × 10.4 × 11.1 cm mass‐like lesion in the left hypochondrium, with the remaining abdominal viscera unremarkable.

CECT of the abdomen revealed a large, well‐defined, heterogeneous mass measuring 10.0 × 11.0 × 14.0 cm within the left upper abdominal mesentery, closely related to the proximal jejunal mesentery and lying anterior to the left kidney. It contained macroscopic fat, dense calcifications with tooth‐like structures, and osseous elements with attenuation of up to 1450 Hounsfield units, and a well‐formed fetal skeleton was visualized (Figure 1). These findings were considered suggestive of a mature teratoma or FIF. No invasive features, bowel‐wall thickening, inflammatory changes, ascites, lymphadenopathy, or evidence of metastatic disease were identified, and the remaining solid organs were unremarkable.

**FIGURE 1:**
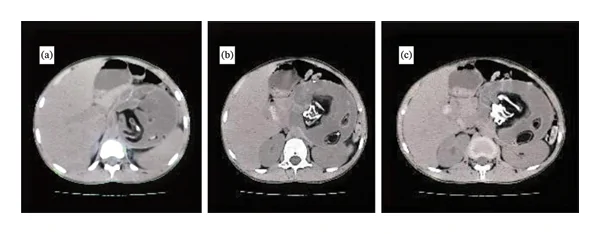
Axial contrast–enhanced CT images demonstrating a large heterogeneous mesenteric mass suggestive of fetus in fetu: (a) a distinct, well‐formed vertebral body, a characteristic finding of fetus in fetu; (b) a predominantly cystic and solid mass containing central calcified and bony structures, with significant mass effect and anterior displacement of the bowel loops; and (c) complex internal architecture with multiple rudimentary long bones, suggestive of malformed upper‐ and lower‐limb structures.

At admission, an infected cystic lesion, a mesenteric or retroperitoneal tumor, and a teratoma were considered in the differential diagnosis. Repeat ultrasonography redemonstrated the mass in the left hypochondrium and showed a dilated bowel loop measuring up to 25 × 16 mm in cross‐section, with loop dilatation extending up to 35 mm. The 25 × 16 mm measurement refers to the dilated bowel loop; the mass itself measured 8.5 × 10.4 × 11.1 cm, as per the original radiology report. The patient was kept nil per os; a nasogastric tube was placed; and intravenous fluids, ceftriaxone, and metronidazole were commenced.

Laboratory investigations showed a hemoglobin of 11.9 g/dL, which decreased to 10.5 g/dL over the following 48 h. Leukocyte and platelet counts, liver and renal function tests, electrolytes, and coagulation profile were normal. HIV, hepatitis B, and hepatitis C serology were negative. Serum alpha‐fetoprotein (< 1.03 ng/mL) and beta‐human chorionic gonadotropin (< 1.0 mIU/mL) were within normal limits.

After informed consent was obtained, an exploratory laparotomy was performed under general endotracheal anesthesia. Through a midline incision, no free intraperitoneal fluid or contamination was found, and all intraabdominal viscera appeared normal apart from a bulge in the left upper abdomen. On incising the overlying peritoneum, a well‐encapsulated, highly vascular cystic mass was found within the mesentery of the proximal jejunum in the left upper abdomen, adherent to major vessels and displacing the small bowel to the right without causing obstruction (Figure 2).

**FIGURE 2:**
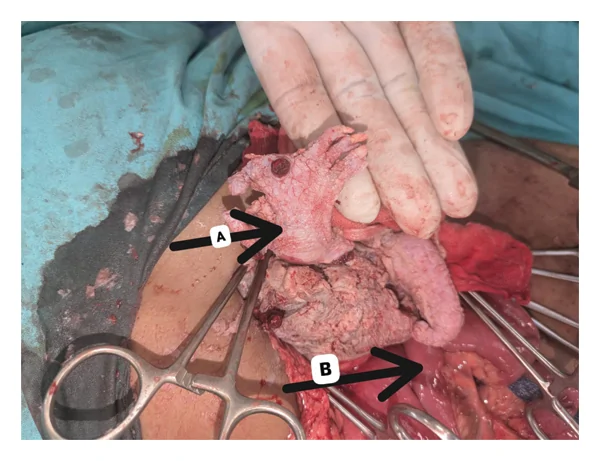
Intraoperative photograph showing surgical mobilization of a large, vascularized, partially necrotic mesenteric mass: (A) fetiform mass with surrounding fibrous adhesions and (B) displaced small‐bowel loops.

On puncture, the cyst yielded thick, purulent‐appearing material, which was completely aspirated; a sample of the aspirate was collected for microbiological culture but was not adequately preserved during transfer, so it could not be cultured and no microbiological result is available. Beneath the cystic component, well‐formed fetal‐like structures with discernible limbs, hair, and nails were identified, and palpation revealed a well‐formed vertebral column, satisfying the Willis criteria and supporting a presumptive diagnosis of FIF.

The mass was carefully dissected from the surrounding mesenteric tissue; its vascular pedicle was ligated and divided, and the mass was excised completely with its capsule, with no residual tissue and no vascular injury (Figure 3). Estimated blood loss was 300–400 mL. The operative field was irrigated with normal saline, retroperitoneal and pelvic drains were placed, and the abdomen was closed in layers. The procedure was well tolerated.

**FIGURE 3:**
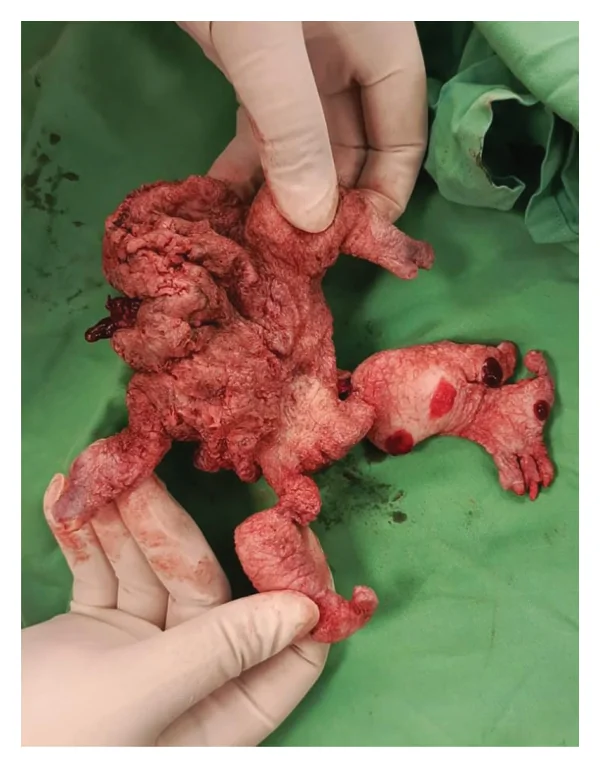
Gross specimen of the resected mesenteric mass, revealing a distinct fetiform structure with a central trunk and well‐formed upper‐ and lower‐limb–like appendages.

The histopathological specimen was, however, inadequately fixed during transfer and was received by the laboratory in a condition unsuitable for analysis. Likewise, the microbiological sample described above could not be cultured because of inadequate preservation. Consequently, neither histopathological nor microbiological confirmation could be obtained, and the diagnosis remains presumptive, resting on radiological and intraoperative findings alone (see Discussion and Limitations).

Postoperatively, the child remained hemodynamically stable and did not require intensive care. He received one unit (500 mL) of packed red blood cells. Hemoglobin was 11.9 g/dL on admission and 10.5 g/dL 48 h later; estimated intraoperative blood loss was 300–400 mL. The nasogastric tube was removed on Postoperative Day 4, and oral feeding resumed the following day. The retroperitoneal drain (250 mL of serosanguineous fluid) was removed on Day 4, and the pelvic drain (550 mL) was removed on Day 7. Other than surgical‐site pain, no complications were documented, and the patient was discharged on June 27, 2026, in stable condition, ambulatory, and tolerating oral feeds without abdominal complaints.

At the first postoperative follow‐up visit on Aug 12, 2026, the patient was clinically well. At a second, telephone follow‐up on Sep 03, 2026, his father reported that the child remained asymptomatic, with no abdominal or surgical‐site pain, vomiting, abdominal distension, loose stools, or constipation, and that the wound was clean. The last available follow‐up (Sep 03, 2026) was approximately 10 weeks after discharge, and longer surveillance is therefore required. A timeline of presentation, investigation, management, and follow‐up is given in Table 1.

**TABLE 1:** Timeline of clinical presentation, diagnostic evaluation, surgical management, and postoperative follow‐up.

Date/timepoint	Event	Key finding/outcome
June 15, 2026	Imaging	Large mass with fetal skeleton; FIF vs. teratoma

June 16, 2026	Admission	Abdominal pain, fever, weight loss

June 16–18, 2026	Investigations	Normal tumor markers; mild anemia

June 2026	Surgery	Exploratory laparotomy and complete mass excision

Postoperative days 4–7	Recovery	Nasogastric tube and drains removed; feeding resumed

June 27, 2026	Discharge	Stable and ambulatory

Aug 12, 2026 (1st follow‐up visit)	Outcome (follow‐up)	Asymptomatic, with a clean wound
Sep 03, 2026 (2nd telephonic follow‐up)		

## 3. Discussion

FIF has an estimated incidence of 1 in 5,00,000 births, and fewer than 200–250 cases have been reported worldwide [1]. The great majority of reported cases occur in males, with only a small number described in females [5]. Although FIF is most often diagnosed in infancy, later presentations have occasionally been documented; our patient’s presentation at 10 years of age is unusual, though not unprecedented, as isolated cases have been reported into adolescence and adulthood [1, 5].

Two principal theories have been proposed to explain the pathogenesis of FIF. The monozygotic twin theory holds that FIF represents a diamniotic, monochorionic, monozygotic twin that becomes incorporated into its co‐twin after anastomosis of the vitelline circulation during early embryogenesis—an abnormal, unequal twinning event in which one twin is engulfed by the other [8]. The teratoma‐spectrum theory instead proposes that FIF is not a twin at all, but a highly differentiated, organized form of mature teratoma arising from pluripotent embryonic cells within the host [9]. Neither theory fully accounts for all reported cases, and the relationship between FIF and highly organized teratoma is best regarded as an area of ongoing debate rather than a settled distinction [2, 3].

Patients with FIF commonly present with nonspecific symptoms such as abdominal discomfort, nausea, vomiting, or constipation, related to mass effect rather than to any intrinsic malignant behavior [10].

Several imaging and clinical features are traditionally used to favor FIF over teratoma, although none is individually definitive. FIF is most often located in the retroperitoneum, though an intramesenteric position, as in our patient, has also been described and lies within the same developmental territory; teratomas, by contrast, although capable of arising almost anywhere, are more frequently midline or found in the pelvis, sacrococcygeal region, or gonads, and abdominal teratomas outside these sites are comparatively uncommon [11, 12]. In our patient, the mass arose within the proximal jejunal mesentery in the left upper abdomen (left hypochondrium), as shown on both CT and at laparotomy, away from the midline, a lateral, off‐midline distribution more in keeping with FIF, although this feature alone does not exclude a teratoma. Similarly, a markedly elevated alpha‐fetoprotein is more characteristic of immature or malignant teratomas containing yolk‐sac elements, whereas a normal level, as in our patient, is supportive of, but not diagnostic for, FIF, as it does not exclude a mature or well‐differentiated teratoma [12]. CECT is valuable for preoperative planning, as it can define the vertebral axis, limb buds, fat and bone content, vascular supply, and relationship to adjacent structures, thereby narrowing the differential diagnosis and guiding the surgical approach, even though imaging alone cannot substitute for histopathological confirmation [12].

The Willis criteria remain the principal diagnostic benchmark for FIF: the presence of a vertebral axis with an organized arrangement of limbs and organs is considered to favor FIF over teratoma [7]. A vertebral column and associated fat can often be identified on CT, as in our case, where imaging showed macroscopic fat, dense tooth–like calcifications, and osseous elements, and a well‐formed vertebral column with limb‐like structures bearing hair and nails was identified intraoperatively. It should be emphasized, however, that a minority of otherwise genuine FIF cases lack a demonstrable vertebral column, so its presence supports, but its absence does not exclude, the diagnosis.

Immature or malignant teratomas are well recognized, whereas FIF is generally regarded as a benign, non‐neoplastic anomaly; nonetheless, malignant transformation arising from a genuine FIF has been documented, albeit in a single reported case, and long‐term surveillance after excision has therefore been recommended even when the mass has been completely excised and behaves clinically as benign [13, 14]. Recurrence of FIF itself is rare after complete excision, as achieved in our patient, but the possibility of late recurrence, or of an initially unrecognized malignant component, underscores the value of continued clinical follow‐up rather than a single postoperative review [14, 15].

### 3.1. Limitations

A central limitation of the present case is that histopathological confirmation could not be obtained, as the resected specimen was inadequately fixed before it reached the laboratory; the microbiological sample was likewise inadequately preserved and so could not be cultured. Histopathology remains the reference standard for distinguishing FIF from a highly differentiated teratoma, since it can identify organized, organ‐specific tissue and an axial skeleton, whereas immature neuroepithelial or other embryonal elements would instead point toward teratoma. In its absence, our presumptive diagnosis rests on the convergence of radiological findings (fat, calcification, and an organized skeleton), intraoperative gross findings (a fetiform structure with limbs, hair, nails, and a vertebral column, satisfying the Willis criteria), and normal tumor markers (supportive but not diagnostic). This approach is not without precedent: a comparable case in which histopathology could not be performed for logistical reasons was nonetheless accepted as FIF on the basis of imaging and gross findings alone [7]. We nonetheless acknowledge that, without histology, a very highly differentiated fetiform teratoma cannot be entirely excluded with certainty, and we highlight the failure of specimen and sample preservation as an avoidable limitation that should prompt closer attention to specimen‐handling protocols in similar cases.

Treatment of FIF is surgical, and complete excision, as achieved in our patient, is generally curative. Even when teratoma remains part of the differential diagnosis, surgical excision is the appropriate management, since it is both diagnostic and therapeutic [15]. Given the reported, albeit rare, possibility of malignant recurrence, we recommend a period of structured clinical follow‐up rather than a single postoperative visit, particularly in cases such as ours where histopathological confirmation is unavailable. The follow‐up available for our patient is short (the last contact, on Sep 03, 2026, was about 10 weeks after discharge) and cannot exclude late recurrence or malignant transformation. We therefore propose long‐term surveillance for this patient, comprising clinical review with abdominal ultrasonography and serum alpha‐fetoprotein at 3, 6, and 12 months after surgery and then annually for at least 5 years, with cross‐sectional imaging if symptoms, a palpable mass, or a rising marker level occur.

## 4. Conclusion

FIF is a rare condition that most clinicians will encounter, if at all, only once in their careers, and it can be difficult to diagnose with confidence. Our case illustrates that FIF should remain in the differential diagnosis of a complex abdominal mass even outside the infantile age group, and that a presumptive diagnosis supported by the Willis criteria, radiological findings, and intraoperative gross morphology is reasonable when histopathological confirmation is unavailable, provided this limitation is explicitly acknowledged and the diagnosis is not presented as definitive. Complete surgical excision remains curative in most cases, but given the rare potential for malignant transformation, sustained clinical follow‐up is warranted, and longer surveillance is needed in our patient.

## Funding

No funding was received for this work.

## Ethics Statement

No institutional ethics committee approval was required for this individual case report; patient confidentiality and anonymity were maintained throughout.

## Consent

Written informed consent was obtained from the patient’s parent/guardian for publication of this case report, including all clinical details and images, in accordance with the journal’s policies.

## Conflicts of Interest

The authors declare no conflicts of interest.

## Supporting Information

Additional supporting information can be found online in the Supporting Information section.

## Supporting information


**Supporting Information** CARE checklist (2013) of information to include when writing a case report.

## Data Availability

The data supporting the findings of this case report are available from the corresponding author upon reasonable request.
